# Intensity of Caring About an Action’s Side-Effect Mediates Attributions of Actor’s Intentions

**DOI:** 10.3389/fpsyg.2018.01329

**Published:** 2018-08-03

**Authors:** Yu Liao, Yujia Sun, Hong Li, Gedeon O. Deák, Wenfeng Feng

**Affiliations:** ^1^Department of Psychology, School of Education, Soochow University, Suzhou, China; ^2^Brain Function and Psychological Science Research Center, Shenzhen University, Shenzhen, China; ^3^Department of Cognitive Science, University of California, San Diego, San Diego, CA, United States

**Keywords:** attribution bias, intentionality, outcome, norm, side-effect effect, subjective values

## Abstract

The side-effect effect (SEE) is the observation that people’s intuition about whether an action was intentional depends on whether the outcome is good or bad. The asymmetric response, however, does not represent all subjects’ judgments (Nichols and Ulatowski, 2007). It remains unexplored on subjective factors that can mediate the size of SEE. Thus, the current study investigated whether an individual related factor, specifically, whether adults’ intensity of caring about an outcome of someone’s actions influences their judgments about whether that person intended the outcome. We hypothesized that participants’ judgments about fictional agents’ responsibility for their action’s side-effects would depend on how much they care about the domain of the side-effect. In two experiments, the intensity of caring affected participants’ ascription of intention to an agent’s negative unintended side-effect. The stronger ascription of intentionality to negative than positive side-effects (i.e., the SEE; Knobe, 2003) was found only in domains in which participants reported higher levels of caring. Also, the intensity of caring increased intentionality attributions reliably for negative side-effects but not for positive side-effects. These results suggest that caring about a domain mediates an asymmetrical ascription of intentionality to negative more than positive side-effects.

## Introduction

Intention attributions carry great importance within contexts ranging from legal systems to schools, families, and informal social groups (Darley and Shultz, 1990; Ohtsubo, 2007; Lagnado and Channon, 2008; Young and Saxe, 2009). However, it is ambiguous whether an outcome was carried out intentionally or not in some situations, especially when an action causes multiple outcomes (even if only one was intended). Additional unintended consequences, or side-effects, can be positive or negative (or both). Previous studies have found that the intentionality of positive and negative side effects was attributed differently.

Consider a case in which a CEO of a company decides to begin a profitable project that is also predicted to harm the environment. Would people believe that the environmental harm is intentional? According to Knobe (2003) and others (Nichols and Knobe, 2007; Mallon, 2008; Phelan and Sarkissian, 2008), roughly 80% of adults (in WEIRD samples; Henrich et al., 2010) agree that the CEO intended to cause the damage. Interestingly, however, if the unintended side-effect of the project is a benefit to the environment, only ∼20% of adults judge that the CEO intended to help the environment. This striking asymmetry has since been replicated in a variety of scenarios in addition to the original CEO scenario (e.g., Knobe and Mendlow, 2004; Malle, 2006; Nadelhoffer, 2006; Pellizzoni et al., 2009; Vonasch and Baumeister, 2017), and is termed the side-effect effect (SEE).

Because the scenario and questions are identical except for the ethical valence of the side-effect, researchers think that this asymmetry shows that moral judgments influence attributions of intentionality – thus, reasoning about these mental state dimensions is interdependent (Adams and Steadman, 2004a,b; Knobe, 2006; Nadelhoffer, 2006; Nado, 2008). Morality-based interpretations, however, have been challenged by studies showing that the response asymmetry between good and bad outcomes is not limited to situations that elicit moral judgment (Machery, 2008; Guglielmo and Malle, 2010; Uttich and Lombrozo, 2010; Rakoczy et al., 2015). Also, the asymmetry is preserved even when a harmful side-effect has its own morally desirable side-effect (e.g., the CEO’s profitable but environmentally unfriendly plan ends up breaking Nazi laws; Nichols and Knobe, 2007; Beebe and Buckwalter, 2010; Sripada, 2012).

Several recent accounts argue that explicit intentionality judgments are not necessary to elicit the SEE: adults also show the SEE when asked to judge whether the CEO’s actions were “known,” “decided,” “advocated,” etc (Pettit and Knobe, 2009; Beebe and Buckwalter, 2010). This suggests that the valence of the side-effect does not only affect judgments of intentionality: it has a broader effect on epistemological evaluations. One account of these results relates them to a norm violation bias: people tend to judge an effect as intended if the preceding action violated a norm because behaviors that conform to norms are less informative about the actor’s underlying mental states or traits than behaviors that violate norms. This asymmetry in inferring non-normative mental states or traits generates asymmetric SEE judgments about either intentionality or knowledge (Holton, 2010; Uttich and Lombrozo, 2010).

Although the norm violation view provides an explanation for asymmetric judgments of positive versus negative outcomes, it does not explain the individual difference in SEE. For example, in the CEO scenarios described above, Nichols and Ulatowski (2007) found that approximately one-third of adults exhibited SEE, one-third judged that neither positive nor negative outcomes were intentional, and one-third judged both outcomes as intentional. The authors attribute this result to individual differences either in people’s interpretation of “intentional” or their concept of intention (Nichols and Ulatowski, 2007). This hypothesis is plausible, but they do not predict the SEE for epistemological or other concepts (e.g., “knowing,” “advocating”). Nonetheless, the finding implies that whether SEE is based on moral judgments or norm violations, only a minority of adults believe that negative events are more intended (or norm-violating) than positive events. Consistently, a recent study reported that for German speakers, the SEE depends on the specifics of the scenario content and is difficult to obtain outside the original CEO scenario (Lau and Reisenzein, 2016).

It is currently unknown what factors account for individual differences in asymmetrical judgments of moral (or norm) violations. In everyday life individuals might follow some moral norms tightly (i.e., taboo topics in formal conversation), and others loosely (i.e., social norms about not interrupting a speaker). This variability might, however, partly depend on our specific attitude toward the norms in question. Violation of norms about which we care greatly, or highly value, might be more salient than violations about norms about which we are “looser” or less concerned. The degree of care or value might modulate the availability or salience of norm violations, and this might in turn influence causal inferences. If this were correct, then violation of high-care norms would more readily elicit mental state or trait ascriptions, and thus a greater asymmetry in moralistic inferences about positive and negative side-effects. That is, the SEE might be mediated by the subjective intensity of an individual’s concern about a particular kind of outcome.

The concept of “caring” (as a value rather than a practice) has been relegated to ethical and practical philosophy (e.g., Held, 2007; Slote, 2007), where it is described in reference to a person’s subjective emotional and moral investment in an object, person, topic, or domain. By contrast, the psychological literature has largely focused either on individual valuation of personal outcomes or on relations between an individual’s attitudes (often treated as an unanalyzed factor that conflates caring/concern with other evaluative dimensions) and their beliefs and/or (real or imagined) actions (e.g., Stern et al., 1995). Yet relatively little attention has been paid to how caring^1^ intersects with reasoning (i.e., inference, decision-making, and related biases). The current study investigates one way in which these factors might interact. Specifically, we hypothesized that subjective level of caring about the recipient or domain of an effect might moderate the SEE bias.

To explore individual differences in attributions of intentionality, we investigate how individuals’ degree of emotional concern – hereafter called Intensity of Caring, or IoC – about a domain relates to their SEE for outcomes affecting that domain. In two experiments we asked individual participants how much they cared about specific affected objects or domains, and then measured SEE for those objects or domains. Experiment 1 compared participants’ judgments of intentionality for scenarios about an object or domain that each participant cared most about versus one that they cared least about, based on individuals’ prior ratings of nine possible objects or domains (e.g., the environment; historic sites; corporate relations). Experiment 2 used the standard CEO test but assessed how much each participant cared about the environment. This approach minimized possible unintended differences between high- and low-care scenarios and provided data that could be compared directly to previous studies. Responses to high-care versus low-care scenarios, with both positive and negative side effects, were compared in both experiments.

## Experiment 1

### Method

#### Participants

The final sample contains 119 college students (81 females) aged 18–31 years (mean = 21.2 years) participated in the study after providing informed consent as stipulated by the ethical committee of Southwest University (Chongqing, China). All participants were randomly assigned to one of two testing groups: 53 participants (40 females, age = 18–27 years, mean = 21.7) participated in the positive side-effect group and 66 participants (41 females, age = 18–31, mean = 21.7) participated in the negative side-effect group. Another 11 participants were eliminated from the final analysis due to inconsistent responses to the *pre-care questions* and the *post-care questions* (see below). Participants received RMB ¥5 for participating. All participants had normal or corrected-to-normal vision and were naive to the purpose of the experiment.

#### Materials and Procedure

A 2 (side effect valence: positive vs. negative) × 2 (intensity of caring: high vs. low) mixed design was implemented. Valence was a between-subject factor, and IoC was a within-subject variable.

Because one specific side-effect could elicit considerably different IoC across individuals, it is it is necessary to evaluate individual participants’ attitudes in order to assign high-/low-IoC test scenarios without consulting with participants. Thus, to maximize possible differences in high-IoC vs. low-IoC conditions within-subjects, and to increase reliability within and between subjects, we implemented pre-test IoC and post-test IoC questions. The pre-test questions were designed for selecting high-/low-IoC test scenarios appropriate for each individual; the post-test questions were intended to check reliability. For the pre-test IoC questions, a list of nine critical events (**Table 1**) was shown to participants at the beginning of the task. Each of the critical events was a brief description of the negative side-effect from one of the nine candidate scenarios pairs; each pair included a positive and a negative side-effect version. Participants were asked, “Among these nine events, which one would you care about the [most/least] if it happened?” Order of most/least questions was counterbalanced. Participants’ answers determined which side-effects would be used as the full high- or low-IoC test scenarios. Post-test IoC questions were also presented following the test questions: again participants were asked to evaluate how much they cared about the side-effect on a 5-point Likert scale ranging from “do not care at all” to “care very much.” Participants were eliminated if their pre-test and post-test responses were inconsistent (i.e., the high-care scenario was rated <3 in the post-test or the low-care scenario was rated >3; this eliminated 11 out of 130 participants).

**Table 1:** Summary of frequencies for selecting high- and low-care events.

Critical events listed in the care-level question before the test scenarios (presented randomly in the test but ordered by frequency of selection). Only negative outcomes are shown, for brevity.	Care most		Care least	
	*n*	%	*n*	%
1. Your job opportunities are taken away.	52	48.1	1	0.9
2. China’s global reputation is harmed.	27	25	1	0.9
3. The environment is harmed.	14	13	5	4.6
4. Some ancient historical sites are destroyed.	4	3.7	1	0.9
5. Your MP3 player suffers from a virus.	3	2.8	5	3.7
6. A new medicine is marketed with more harmful side effects.	2	2.8	2	1.9
7. A stranger’s public reputation is harmed.	3	2.8	20	18.5
8. The income of workers from a company decreased.	2	1.9	20	18.5
9. The relationship between company A and company B is harmed.	0	0	54	50

The nine pairs of scenarios included the standard SEE CEO/environment scenario and eight other scenarios, written to match the structure of the standard scenario (Knobe, 2003). In all scenarios an agent chose to perform action A with the intention to achieve outcome O, and with the knowledge that it would also cause side-effect S. Each scenario had two possible side-effects with opposite valence: positive (S+) or negative (S-). For example, in one scenario an agent chose to publish a piece of news (A), intending to increase the newspaper’s circulation (O), but knowing that it would help (S+) or harm (S-) China’s global reputation. Each pair of scenarios used the same phrasing. All scenarios were presented in Chinese. The full text is available from the corresponding author.

The test question was presented at the end of each scenario. Participants were asked whether the agent intentionally caused the side-effect [e.g., *Did the editor intend to harm China*’*s global reputation? Yes (1) or No (2)?*]. Participants responded by pressing the corresponding numeric key on the keyboard. All materials were presented on a computer screen using E-Prime 1.6 software (Psychology Software Tools, Inc., Sharpsburg, PA, United States). All participants were tested in the same behavioral testing room. Their responses to each question were recorded during the session.

### Results

Participants’ responses to the pre-test IoC questions are summarized in **Table 1**. As predicted, the nine critical events elicited a range of IoC levels across individuals. Post-test IoC questions further confirmed that participants’ subjective IoC level for the high-care scenario was significantly higher (mean = 3.65; *SD* = 0.60) than for their low-care scenario (mean = 2.21; *SD* = 0.57); *t*_(107)_ = 4.29, *p* < 0.001.

Because participants’ responses to both high- and low-IoC scenarios were binary outcomes (i.e., Intended: Yes or No), and the two scenarios were administrated within subjects, we employed binary generalized estimating equations (GEE, SPSS22) models to investigate the main effect of valence and subjective care level, as well as their interactive effect on participants’ intention ascription. GEE is an extension of the generalized linear model for regressions involving observations that arise from repeated within-subject measurement, and it allows for binary dependent data.

**FIGURE 1:**
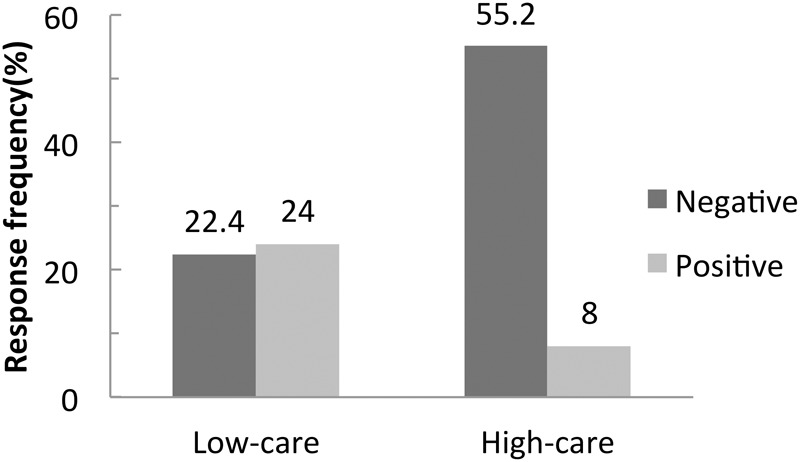
Proportions of intention ascription for negative and positive side effects, for scenarios that elicited highest or lowest concern.

Generalized estimating equations revealed that whereas the main effect of outcome valance was not significant, but the main effect of IoC level was significant [*B* = 1.29, *SE* = 0.66, *p* < 0.001, Exp(B) = 3.60, CI 95% -0.008; 2.59]. More crucially, there was an interaction between outcome valence and IoC level [*B* = 2.739, *SE* = 0.77, *p* < 0.001, Exp(B) = 15.47, CI 95% 1.23; 4.25]. Pair-wise comparisons indicated that intentionality was attributed to **S-** scenarios in a high-IoC domain significantly more often (55.2%, or 32 out of 58, see **Figure 1**) than to **S+** scenarios in a high-IoC domain (8%, or 4 out of 50); *X*^2^_(1)_ = 26.89; *p* < 0.001. This result illustrates the standard SEE asymmetry. By contrast, for low-IoC scenarios, there was no difference in the proportion of participants attributing intentionality to **S-**(22.4%, or 13 out of 58) vs. **S+**(24%, or 12 out of 50, see **Figure 1**) outcomes. Thus, the SEE asymmetry did not generalize to domains for which participants professed little care or interest.

In addition, pair-wise comparisons indicated a higher rate of intentionality ascription for S- scenarios in high-IoC domains than in low-IoC domains; *X*^2^_(1)_ = 13.107; *p* < 0.001. Conversely, there was a lower rate of intentionality ascribed to S+ scenarios in high-IoC domains than in low-IoC domains; *X*^2^_(1)_ = 4.76; *p* < 0.05. This means that if participants cared more about a domain, they were more likely to judge an S**-** as intentional, but less likely to judge an S+ as intentional. These results support the hypothesis that subjective care modulates subjects’ ascription of intentionality for positive vs. negative side effects.

## Experiment 2

Experiment 2 focused on the widely used CEO/environment test and tested whether the SEE effect varied according to how much participants claimed to care about the environment. We hypothesized that those who care more should show a stronger effect. This might contribute to an explanation of the individual differences in SEE asymmetry reported by Nichols and Ulatowski (2007).

In Experiment 2 participants heard both the **S+** and **S-** versions of the standard CEO scenario: that is, the valence of the side-effect was varied within-subjects. Although this might weaken the SEE asymmetry because some participants might notice and correct their own inconsistency across responses, it nonetheless also provides a stringent test of the SEE, and holds sampling variance constant. Subjective level of care about the environment was a random variable based on rank order (see below).

### Method

#### Participants

Eighty-one new participants (45 females, age = 18–31 years, mean = 21.3) were recruited from Southwest University, Chongqing, China, as in Experiment 1. Participants received RMB ¥5 for their participation.

#### Materials and Procedure

All participants received both **S+** and **S-** versions of the standard CEO/environment scenario. Half of the participants were randomly chosen to complete the **S+** scenario first; the rest completed the **S-** scenario first. In addition, to assess individual IoC about the environment, participants ranked all nine critical **S-** events used in Experiment 1 (see **Table 1**). The instructions were: “Please read the following events and rank them from the one you would care about the most to the one you would care about the least if it happened.” Materials were administered in an untimed group pen-and-paper procedure. We used the ranking approach instead of simply asking how much the participant cared about the environment because participants might have considered it socially unacceptable to explicitly state that they do not care about the environment. Therefore, a Likert scale might bias participants against making low ratings whereas ranking a list of events relies on un-anchored comparisons that might not elicit social expectations.

### Results

**FIGURE 2:**
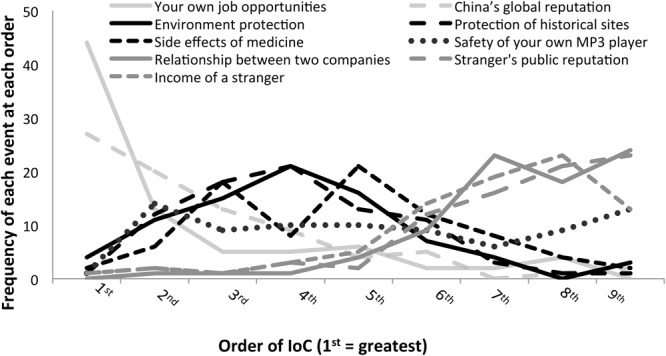
Frequency of reported IoC for all nine critical events.

Participants showed different care rankings for each of the nine events (**Figure 2**). The average order of the ranking from high to low is: your own job opportunities; China’s global reputation; protection of historical sites; environment protection; side effects of medicine; safety of your MP3 player; relationships between two companies; a stranger’s public reputation; a stranger’s income.

Among these, as expected, environment protection (the solid black line) elicited distributed levels of caring, with an average rank of 5.88 out of 9 (1 = lowest care), *SD* = 1.81. This distribution allows us to further examine the relationship between subjective IoC and ascriptions of intentionality of S**-** and S+ side-effects in the standard CEO test.

The results replicated the SEE asymmetry reported in previous studies (Knobe, 2003; Nichols and Ulatowski, 2007; Mallon, 2008; Phelan and Sarkissian, 2008). Significantly more participants claimed that the CEO intentionally harmed the environment (35.8%, 29 out of 81) than intentionally helped the environment (8.6%, 7 out of 81); *X*^2^_(1)_ = 17.29; *p* < 0.001. This is noteworthy because, in the within-subjects design, participants easily might have modified their second response to maintain internal consistency or to correct a biased response.

**FIGURE 3:**
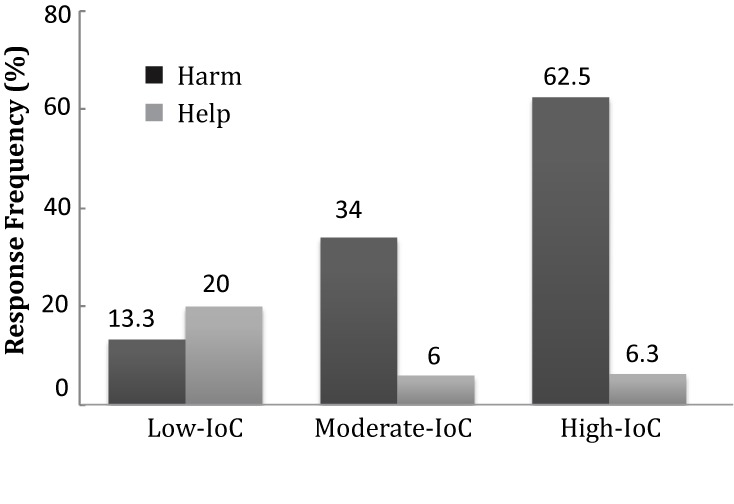
Proportions of intention ascriptions for harmful (S–) and helpful (S+) by participants with different IoC levels.

To further examine how subjective care about the domain moderated intention ascription, we conducted separate binary logistic regression analyses of for the help (S+) and harm (S**-**) conditions data. The nine ranks of relative IoC were collapsed into three levels prior to regression analysis, to facilitate an intuitive understanding. These were defined as low-IoC (*n* = 15, ranks 1–4, mean = 2.93, *SD* = 1.28), moderate-IoC (*n* = 50, ranks 5 or 6, mean = 5.6, *SD* = 0.49), and high-IoC (*n* = 16, ranks 7–9, mean = 7.65, *SD* = 0.71). The dividing points were chosen so that the moderate group reflected the grand mean (5.88), and so that each group was large enough to permit comparison. Proportions of ascribed intentionality by the low-, moderate-, and high-IoC groups, respectively, were 20.0, 6.0, and 6.3% for the helpful (S+) side-effect, and 13.3, 34.0, and 62.5% for the harmful (S**-**) side-effect (**Figure 3**).

In the S**-** condition, a binary logistic regression showed that participants with high environmental IoC ranks were ∼2.7 times more likely to claim that the CEO intended the harmful side-effect (β = -0.72; OR = 2.738, *p* < 0.001; CI 95% 1.33; 5.63). In the S+ condition, a binary logistic regression showed that environmental IoC rank did not predict ascription of intentionality (β = 1.01; OR = 0. 48, *p* = 0.25; CI 95%.13; 1.67). This pattern, like the results of Experiment 1, shows that participants’ subjective intensity of caring moderated their tendency to ascribe intentionality to the negative versus positive side-effects of an agent’s action.

## General Discussion

The current study investigated whether a person’s IoC or caring about a domain mediates their judgments that the positive or negative side-effects of another’s actions are intentional. Although *caring* has been discussed from the perspective of moral philosophy (e.g., Held, 2007), it has not been the focus of many psychological studies of moral reasoning. Experiments 1 and 2 compared participants’ judgments for causal side-effect scenarios associated with high- or low-IoC. In both experiments, participants responded differently to low- and high-IoC scenarios. The asymmetrical SEE, or ascribing intentionality more to negative than positive side-effects (e.g., Knobe, 2003; Nichols and Ulatowski, 2007; Mallon, 2008; Phelan and Sarkissian, 2008), was found if participants reported moderate- to high-care about the domain of the scenario. No such asymmetry was obtained if participants did not care much about the domain. The results suggest that the SEE is mediated by an individual’s subjective care level.

In addition, our results indicated that the subjective IoC differentially modulates intention-attribution for positive and negative side-effects. In Experiment 1, participants showed a relative increase in intention-ascription for negative side-effects and a decrease for positive side-effects for high-IoC topics, relative to low-IoC topics: that is, the bias reflected a shift of causal inferences for *both* positive- and negative-side-effect, but in opposite directions. This finding can assimilate prior results: for example, Guglielmo and Malle (2010) reported a stronger asymmetry in the CEO scenario (87% vs. 20% “intended” responses for help and harm versions, respectively) than in a scenario that involved breaking (or conforming with) a social norm (i.e., a party’s dress code: 64% vs. 35% “intended” responses). Although the researchers did not assess participants’ level of care, it is likely that most college students care less about breaking a party dress code than about environmental damage. Thus, IoC might explain this result.

Researchers have made considerable effort to explain SEE (for recent reviews, see Cova and Naar, 2012; Sloman et al., 2012), however, so far, no generally accepted explanation has been found. Some researchers have related the SEE to moral reasoning (Knobe, 2006; Nadelhoffer, 2006; Nado, 2008). However, because not all the negative side-effects in our test scenarios are conventionally morally value-laden (e.g., personal job prospects; safety of MP3 player), the results cannot easily be interpreted strictly in terms of morality judgments in inferences about intentionality. Instead, the results fit in a framework of norm-violation (Knobe, 2007). According to this view, any discrepancy between an actual outcome and the predicted or expected outcome based on learned norm would promote intention ascription (Knobe, 2007; Holton, 2010; Uttich and Lombrozo, 2010). This hypothesis can explain the current results for all of our scenarios. In this case, the current results would suggest that an individual’s subjective IoC modulates causal inferences under norm-violation theory.

More specifically, one possible interpretation of the current results is that subjective intensity of care modulates the discrepancy between norm and outcome. That is, either the representation of the norm, or the perceived distance of the scenario outcome from the norm, is modulated by an individual’s degree of care, or emotional investment in, a given domain. For objects or domains of higher subjective care, the represented norm might be more positive, and deviations from the norm might be evaluated as more extreme (i.e., greater “distance” between norm and outcome). Thus, a negative outcome (**S-** or harmful side-effect) is more likely to be tagged as a violation than a positive outcome (**S+** or helpful effect), which will be closer to the (positive-skewed) norm. This tagging of a violation induces participants to generate hypotheses about possible causal elements or forces. Because the scenario names and describes an agent, this agent becomes a highly available entity to fill a slot in the causal model: specifically, an intentional agent that acted with intention to produce the side-effect. By contrast, if the participant cares little about the object or domain, the norm might be more dynamic or context-dependent, or the discrepancy between the norm and the scenario outcome is not salient. Alternatively, the discrepancy might be of insufficient interest to motivate the participant to generate causal hypotheses. Thus, *any* outcome, negative (S**-**) or positive (S+), is less likely to trigger a norm violation. In fact, in the extreme, where a participant has no stable representation of norms, the individual might not clearly represent (or care about) what counts as a remarkably bad or good outcome. Whatever the reason, when a norm violation has not been encoded, it is less likely that the individual will reason about a possible cause. Thus, it is unlikely that discrete causal explanations, including intentionality, will be consciously represented. Instead, perhaps as a default, participants might resort to more “symmetrical” (or non-committal) responses, or unsystematic responses, yielding, on average across these unconcerned individuals, an undifferentiated pattern of answers to questions about negative and positive side-effects.

This account can also accommodate several other findings that point to more than a simple morality-biasing mechanism. There is, for example, evidence that a larger discrepancy from anticipated emotions causes stronger affect (Mellers et al., 1997, 1999). Also, confident prediction of a positive outcome (i.e., a positive outcome norm) results in less pleasure when the positive outcome is attained. This could be because the discrepancy between the actual outcome and the norm is small (McGraw et al., 2004). Similarly, people’s assessments of how successfully they completed a task are modulated by the subjective value or self-relevance of the outcome (Sweeny and Shepperd, 2007). All of these findings suggest that judgment and affect are modulated by the discrepancy between actual outcomes, and expected states or norm (Mellers et al., 1997, 1999; McGraw et al., 2004). Thus, the current account is consistent with claims that the SEE could reflect a general decision-making bias (Cokely and Feltz, 2009), rather than a more specific bias in morality-based reasoning about intentionality.

Nonetheless, the current results raise some questions. First, even in for high-IoC scenarios, the magnitude of the SEE (55.2% vs. 8% intentionality-ascription for negative vs. positive) is not as large as reported in other studies (e.g., ∼80% vs. ∼20% in e.g., Knobe, 2003; Knobe and Mendlow, 2004; Guglielmo and Malle, 2010; Uttich and Lombrozo, 2010). This might be due to the phrasing of the test questions. Using different mental words in test questions can elicit different responses: for example, Knobe (2004) showed that whereas 89% of participants said the CEO was intentionally harming the environment, only 29% said the CEO had the intention to harm the environment. Although we translated and back-translated our materials to ensure the key Chinese verb *youyi* (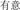) in test question carries the same implications as the key English word *intentionally*, given sometimes subtle language differences in verb meanings (Lucy, 1997), the connotations might not be identical. For this reason, it would be desirable to replicate these findings in different cultural and linguistic groups, using several different mental verbs in each language (as in Knobe, 2006). In addition, it would be illuminating to compare scenarios that imply a wider range of causal agents (sentient and non-sentient), and to collect dependent measures that are more nuanced than a simple yes/no dichotomous choices. The current findings, nevertheless, suggest that subjective value-modulated norms, combined with a human bias to look for available causal forces for highly nor-violating events, can explain the SEE.

## Author Contributions

YL and WF contributed to experimental design, data collection, data analysis, and paper writing. YS contributed to experimental design and data collection. HL and GD contributed to experimental design, data analysis, and paper writing.

## Conflict of Interest Statement

The authors declare that the research was conducted in the absence of any commercial or financial relationships that could be construed as a potential conflict of interest.

## Footnotes

1 For our purposes, we define *caring* as emotion-laden and belief-informed evaluation of, and investment (of time, thought, or material resources) in an object, person, activity, domain, or goal.
